# New Risk Factor-Weighted Clinical Likelihood (RF-CL) in Diagnosis of Chronic Coronary Syndrome in Men and Women: Our First Impressions About the Differences and Opportunities

**DOI:** 10.3390/jcm15082923

**Published:** 2026-04-12

**Authors:** Nurudin Nurutdinov, Anna Akselrod, Andrei Akselrod, Tamerlan Erdniev, Ekaterina Fominykh, Aleksandra Bogdanova, Maria Poltavskaya, Vsevolod Sedov, Nina Novikova, Abram Syrkin, Denis Andreev

**Affiliations:** 1 Department of Cardiology, Functional and Ultrasound Diagnostics, First State Moscow University Named After I.M. Sechenov, Trubetskaya 8/2, 119991 Moscow, Russia; akselrod_a_s@staff.sechenov.ru (A.A.); akselrod_a_b@student.sechenov.ru (A.A.); doc.aabogdanova@gmail.com (A.B.); m.poltavskaya@yandex.ru (M.P.); sedov_v_p@staff.sechenov.ru (V.S.); novikova_n_a@staff.sechenov.ru (N.N.); syrkin_a_l@staff.sechenov.ru (A.S.); andreev_d_a@staff.sechenov.ru (D.A.); 2 Department of Diagnostic Radiology, First State Moscow University Named After I.M. Sechenov, Trubetskaya 8/2, 119991 Moscow, Russia; fominykh_e_v@staff.sechenov.ru

**Keywords:** chronic coronary syndrome, invasive coronary angiography, exercise electrocardiogram testing, non-invasive imaging, Risk-Factor-Weighted Clinical Likelihood (RF-CL), stable coronary artery disease, stress echocardiography, stress myocardial perfusion SPECT, CT coronary perfusion, myocardial ischemia

## Abstract

**Background/Objectives**: To evaluate the Risk Factor-Weighted Clinical Likelihood (RF-CL) model for the diagnosis of chronic coronary syndrome (CCS) in men and women. **Methods**: The study included 222 patients (100 men and 122 women; mean age 64.76 ± 10.3 years) with suspected CCS. Diagnostic evaluation was performed in accordance with the 2024 clinical practice guidelines for stable coronary artery disease developed by the Russian Society of Cardiology. The clinical probability of obstructive coronary artery disease (CAD) was calculated for all patients using the RF-CL model. **Results**: Application of the RF-CL model demonstrated higher probabilities in men than in women (17% [11–27] vs. 6% [4,5,6,7,8,9,10], *p* < 0.001). Moderate CCS probability (RF-CL > 15–50%) was observed in 64% of men and 9.8% of women (*p* < 0.001); low probability (RF-CL > 5–15%) in 30% and 60.7% (*p* < 0.001); very low (RF-CL ≤ 5%) in 6% and 29.5% (*p* < 0.001). The prevalence of typical angina (21% vs. 17.2%, *p* = 0.47), atypical angina (31% vs. 26.2%, *p* = 0.43), and non-anginal chest pain (31% vs. 26.2%, *p* = 0.43) did not differ. Dyspnea was less frequent in men (44% vs. 59.8%, *p* = 0.02). Only 35 (15.8%) patients had indications for invasive coronary angiography (ICA), and significant stenosis (≥70%) was found in 17 patients. According to the ROC analysis, the cutoff value of RF-CL for predicting a positive stress test was 19.5% in men (AUC 0.723; *p* = 0.002), 6.5% in women (AUC 0.479; *p* = 0.852), and 15% in the overall cohort (AUC 0.737; *p* < 0.0001). **Conclusions**: Men with moderate and women with low probability of obstructive CAD are predominantly encountered in clinical practice when the RF-CL model is applied. Our observations have shown that ICA is indicated in relatively few patients and suggest potential overuse of exercise testing regardless of the clinicians’ adherence to the traditional Diamond–Forrester model or the RF-CL algorithm in suspected CCS.

## 1. Introduction

Coronary artery disease (CAD) is the leading cause of death worldwide [1,2,3,4]. According to the GBD study [5], approximately two hundred million cases of CAD were reported in 2019, and the incidence is increasing. Thus, the identification and treatment of all clinical forms of CAD is highly relevant.

In 2019, the European Society of Cardiology released clinical guidelines on the diagnosis and management of patients with chronic coronary syndrome (CCS) [1]. According to these guidelines, exercise stress testing was recommended for patients with a higher pre-test probability of stable angina based on the classical Diamond–Forrester model (DF model). This model considered the patient’s sex, age and clinical presentation. However, despite its simplicity and practical orientation, the DF model did not take into account traditional factors that could refine the estimated likelihood of coronary artery obstruction.

**Figure A1:**
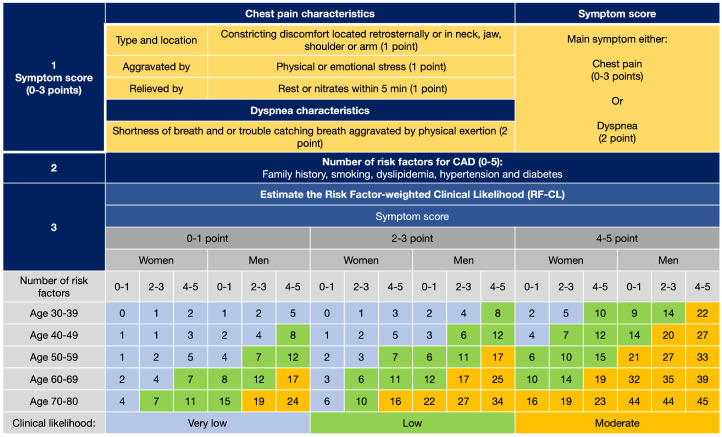
The Risk Factor-Weighted Clinical Likelihood model. CAD, coronary artery disease; RF-CL, Risk Factor-Weighted Clinical Likelihood.

A new model for estimating the clinical likelihood of CCS based on risk factors assessment, the Risk Factor-Weighted Clinical Likelihood (RF-CL) model (Figure A1) was introduced in the 2024 updated guidelines of the European Society of Cardiology. The RF-CL model, unlike the DF model, considers not only the patient’s sex, age and clinical presentation but also determines the contribution of individual risk factors to the probability of CCS. According to the authors of the new guidelines, the traditional DF model tended to overestimate the probability of obstructive CAD in patients with typical angina and high pre-test probability. In such patients, CCS was confirmed by invasive coronary angiography (ICA) only in one-third of cases [2]. Data on the prevalence of certain clinical manifestations of CCS vary according to different studies: typical angina ranges from 10 to 24%, atypical angina up to 78%, non-anginal chest pain about 10%, and exertional dyspnea up to 15%. Several studies have shown clear sex-related differences, non-anginal chest pain being more common in women [6,7,8]. Overall, the 2024 updated guidelines of the European Society of Cardiology suggest a personalized approach to each patient when CCS verification is necessary. These guidelines aim to more precisely identify the subgroup of patients in whom stable CAD is most likely.

The new RF-CL model was developed to identify patients with coronary artery stenosis exceeding 50%. However, its diagnostic performance for detecting myocardial ischemia in men and women has not yet been clearly established [9,10]. Previous studies have shown that approximately 60% of women and 30% of men with stable angina have ischemia with Non-Obstructive Coronary Arteries (INOCA) [11]. These findings highlight a need for a new diagnostic tool for the initial screening of men and women with suspected chronic coronary syndrome.

The heterogeneity of CCS clinical manifestations [3,4] and the low rate of myocardial ischemia confirmed by exercise stress testing suggest that the evaluation of the new RF-CL model with consideration of sex differences is particularly relevant.

Our observational study was conducted between 2024 and 2026 in the Russian Federation, where current clinical guidelines of the Russian Society of Cardiology based on the traditional DF model remain in effect. The investigators acted as independent observers and did not interfere with diagnostic decision-making by treating physicians. Thus, the RF-CL model is not yet included in the official diagnostic algorithm in Russia, although it is increasingly used in clinical practice.

The aim of this study was to evaluate the Risk Factor-Weighted Clinical Likelihood (RF-CL) model for the diagnosis of chronic coronary syndrome in men and women.

## 2. Materials and Methods

**Figure 1:**
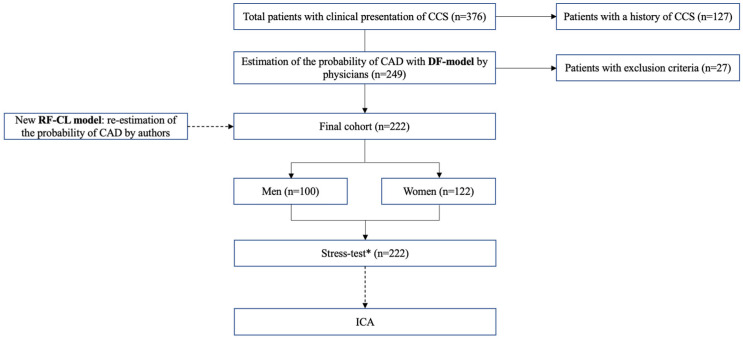
Study protocol. *—stress-test modalities: SPECT, stress echocardiography, exercise ECG. SPECT—single-photon emission computed tomography; ICA—invasive coronary angiography.

Out of 349 patients with suspected CCS, 222 participants (100 men and 122 women) were included in the study. All patients were evaluated at the University Clinical Hospital №1 of Sechenov University from November 2024 to February 2026. The study protocol was approved by the Ethics Committee of Sechenov University (№27-24, dated 7 November 2024). Study design is presented in Figure 1.

Inclusion criteria for this study were: age > 35 years, any clinical manifestations suggestive of CCS lasting more than 4 weeks, and signed informed consent.

Exclusion criteria were as follows: a history of previously confirmed diagnosis of CCS and any other form of CAD, development of any severe comorbid condition or decompensation of a previously known chronic disease, stage 4 or 5 chronic kidney disease (CKD), pregnancy, any previously diagnosed psychiatric disorders, and refusal to participate in this study.

The clinical likelihood of obstructive CAD was calculated using the new RF-CL model, which incorporates family history, smoking status, type 2 diabetes mellitus (T2DM), dyslipidemia, hypertension [2].

According to the 2019 ESC guidelines on chronic coronary syndromes [1], typical angina is defined as constricting discomfort in the chest, neck, jaw, shoulder, or arm that is precipitated by physical exertion or emotional stress and relieved by rest or nitroglycerin. Atypical angina is defined as the presence of two of these characteristics, whereas non-anginal chest pain is defined as pain fulfilling one or none of them.

All patients were referred for stress testing after assessment of the clinical likelihood of CCS. The decision to perform a stress test was made by the treating physician according to the Diamond–Forrester model [4].

A positive stress test was defined as the presence of two or more hypokinetic segments on stress echocardiography, an ischemic burden ≥ 5% on single-photon emission computed tomography (SPECT), or ST-segment depression > 1 mm on exercise ECG testing. A non-informative result was defined as failure to achieve submaximal heart rate due to marked fatigue or as poor image quality on stress echocardiography. Equivocal results included transient ST-segment depression <1 mm without anginal symptoms or the occurrence of angina without definitive ECG changes during exercise ECG testing [12]. Based on the test results and the overall clinical assessment, the treating physician decided whether ICA was indicated.

Data were analyzed using the IBM SPSS Statistics software version 27.0.1. The normality of distribution was assessed using the Kolmogorov–Smirnov test. Clinical and demographic characteristics, risk factors, and data from instrumental investigations and interventions were analyzed using Student’s *t*-test, chi-square test, Fisher’s exact test, and Mann–Whitney U test. The diagnostic accuracy of the RF-CL model was determined using ROC analysis. Differences were considered statistically significant at *p* < 0.05.

## 3. Results

All men (n = 100) and women (n = 122) with suspected CCS (n = 222) were comparable in age and did not differ significantly in the prevalence of hypertension, T2DM, or dyslipidemia. Nevertheless, smoking (*p* < 0.001) and atrial fibrillation (*p* = 0.001) were significantly more frequent in men (Table 1).

The prevalence of associated clinical conditions, such as CKD and chronic heart failure (CHF), was not significantly different between groups. Comparison of clinical presentation between men and women (Table 2) showed similar proportions of typical angina (21% vs. 17.2%, *p* = 0.47), atypical angina (24% vs. 31.1%, *p* = 0.24), and non-anginal chest pain (31% vs. 26.2%, *p* = 0.43). Dyspnea was less frequent in men (44% vs. 59.8%, *p* = 0.02).

Application of the RF-CL model revealed sex-related differences: men had higher clinical likelihood values than women (17% vs. 6%, *p* < 0.001). Similarly, the distribution of CCS probability categories differed: moderate (RF-CL > 15–50%) in 64% of men and 9.8% of women (*p* < 0.001), low (RF-CL > 5–15%) in 30% of men and 60.7% of women (*p* < 0.001), and very low (RF-CL ≤ 5%) in 6% of men and 29.5% of women (*p* < 0.001).

We also analyzed the use of different stress tests in both sexes (Table 3): single-photon emission computed tomography myocardial perfusion scintigraphy, stress echocardiography, exercise ECG testing, and computed tomography (CT) perfusion imaging.

There were no significant sex differences in the frequency of performing SPECT myocardial perfusion (13% in men vs. 11.5% in women, *p* = 0.12). Stress echocardiography was performed more often in women (55% vs. 66.7%), although the difference did not reach statistical significance (*p* = 0.06). Concurrently, exercise ECG testing was performed more frequently in men (45% vs. 30.3%, *p* = 0.025). CT perfusion imaging was the least used method and was performed in only one patient.

Patients were divided into two groups in order to evaluate sex-related differences according to stress test results: group 1 with positive results, group 2 with negative results. Moderate clinical likelihood was more common amongst male patients with positive stress test results: 18 (85.7%) vs. 32 (56.1%) in negative test group (*p* = 0.02). RF-CL values in men were higher in those with positive stress test results (27% vs. 17%, *p* < 0.001) (Table 4).

When men with negative and positive stress test results were compared, the proportion of patients with low clinical likelihood did not differ significantly between the groups (36.8% vs. 14.3%, *p* = 0.10). Similarly, no significant differences were observed in the proportion of men with very low clinical likelihood (7.0% vs. 0%, *p* = 0.18).

Among women, the distribution of RF-CL-based clinical likelihood categories was also similar regardless of stress test results, with no significant differences in the proportions of patients with moderate, low, or very low clinical likelihood.

Taking into account the results of stress tests and the clinical features (positive results, n = 16; doubtful, n = 11; negative results with exertional angina, n = 3; uninformative, n = 5), ICA was performed in 35 (15.8%) patients, significant stenoses (≥70%) were detected in 17 patients (7.7%). Thus, obstructive CAD was confirmed in fewer than 8% of patients evaluated for suspected CCS. Sex differences were also observed at the diagnostic verification stage: ICA was performed more than twice as often in men compared with women (23 (23%) vs. 12 (9.8%), *p* = 0.008).

**Figure 2:**
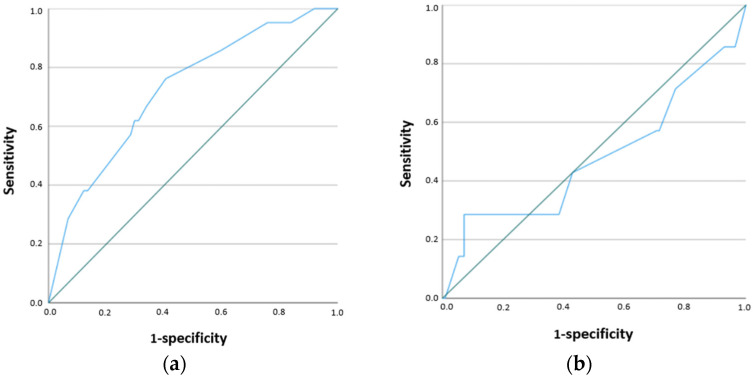
ROC analysis for the RF-CL model in men (**a**) and women (**b**).

**Figure 3:**
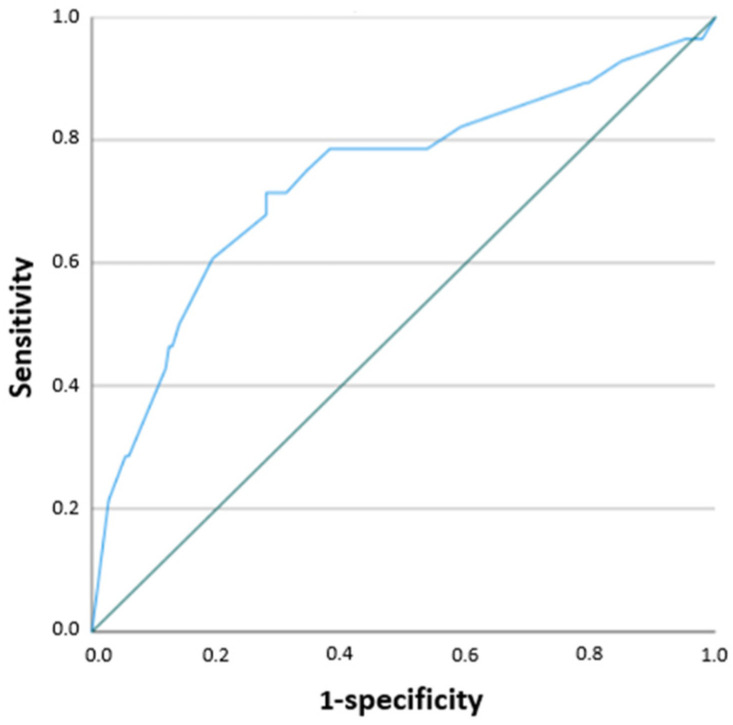
ROC analysis for the RF-CL model in all patients.

According to the ROC analysis, the RF-CL cut-off value for predicting a positive stress test was 19.5% in men (AUC 0.723; *p* = 0.002), 6.5% in women (AUC 0.479; *p* = 0.852), and 15% in the overall cohort (AUC 0.737; *p* < 0.0001) (Table 5, Figure 2 and Figure 3).

## 4. Discussion

We used the new Risk Factor-Weighted Clinical Likelihood model, recommended by the 2024 European Society of Cardiology guidelines on chronic coronary syndrome [2], to estimate the CCS probability in 222 patients. As noted above, a key limitation of our study is the absence of formal implementation of the RF-CL model in current clinical guidelines, which continue to rely on the DF model. During the study period (2024–2026), these guidelines remained in effect in the Russian Federation. Consequently, the RF-CL model is not yet part of the official diagnostic algorithm in routine clinical practice, although it is increasingly used by clinicians. Accordingly, this study has an observational design, as diagnostic decisions were made by treating physicians in accordance with existing guidelines of the Russian Society of Cardiology. The investigators did not influence the diagnostic process or clinical decision-making. Although the cohort was relatively small, the analysis provides clinically relevant preliminary insights for daily practice. Application of the RF-CL demonstrated significantly higher clinical likelihood values in men compared with women (17% vs. 6%, *p* < 0.001), which can be attributed to the relatively higher proportion of positive stress test results in male patients. The distribution of the RF-CL clinical likelihood significantly differed by sex: moderate (RF-CL > 15–50%) in 64% men and 9.8% in women (*p* < 0.001), low (RF-CL > 5–15%) in 30% and 60.7% (*p* < 0.001), and very low (RF-CL ≤ 5%) in 6% and 29.5% (*p* < 0.001), respectively. Using the new RF-CL model, which is becoming an increasingly important tool in cardiological practice, we hypothesized that incorporating traditional CAD risk factors would allow more specific identification of patients with significant coronary artery damage and therefore demonstrate lower probability values of the obstructive CAD compared to the traditional Diamond–Forrester pre-test probability model.

Based on the data we obtained, women with negative and positive stress test results had comparable clinical and demographic characteristics, and their RF-CL clinical likelihood of CCS did not differ between sexes. In contrast, male subgroup of patients differed from each other according to the stress test results: patients with positive stress tests were predominantly in the moderate category (RF-CL > 15–50%). We also understand that the relatively recent (2024) update of CCS management guidelines contributes to the more frequent (about 20%) use of stress testing in patients with very low RF-CL clinical likelihood of CCS. Low adherence of doctors to the new guidelines is also reflected in the minimal use of CT myocardial perfusion, which was performed in only one participant.

Overall, stress testing patterns differed between sexes: stress echocardiography was more frequently performed in women (n = 82, 66.7%; *p* = 0.06), whereas exercise ECG testing was performed predominantly in men (n = 45, 45%; *p* = 0.025). Imaging-based stress techniques are clearly preferable for the diagnosis of CCS; however, in routine clinical practice, exercise ECG testing is still frequently used in patients with low and very low clinical likelihood of CAD. This practice may reflect limited adherence to the new guideline recommendations. Nevertheless, given that the diagnostic accuracy of this technique is higher in men [2,13], the observed sex-specific distribution of stress testing modalities appears justified. Our study also demonstrated a significantly higher prevalence of dyspnea in women than in men (59.8% vs. 44%, *p* = 0.02). These findings differ from those reported in previous studies, where non-anginal chest pain was the most common clinical manifestation of CCS [8].

Among all 222 participants in our study, only 35 (15.8%) patients underwent ICA, as most did not meet the indications criteria according to both the latest Russian Society of Cardiology guidelines on stable coronary artery disease [4] and the 2024 European Society of Cardiology guidelines on the diagnosis and management of CCS [2]. Stenoses >70% were identified in only 17 patients (7.7%), emphasizing the need for further optimization of diagnostic strategies for CCS. Prior studies have shown an increasing prevalence of INOCA in both women (up to 65%) and men (up to 32%), during 1998–2009 [11]. More recent publications also highlight a mismatch between symptoms, ischemia, and anatomical stenosis. For example, in ORBITA-2, anginal symptoms resolved in only 40% of patients who underwent PCI [14]. These findings clearly illustrate the limitations of diagnostic strategies that focus only on stenosis detection.

In the 10-year follow-up of the FAME 2 Trial [15], outcomes were compared between patients undergoing myocardial revascularization and those receiving optimal medical therapy. Subgroup analysis demonstrated the greatest benefit of revascularization in patients with a fractional flow reserve (FFR) below 0.65 (win ratio 1.58; 95% CI 1.15–2.15). Notably, comparable rates of the primary endpoint were observed in patients with stenoses ≥ 70%, which may be considered as an alternative to FFR assessment. In this context, further development and evaluation of non-invasive tools for stratifying patients with stenoses ≥ 70% remains a promising direction for contemporary cardiology. In summary, our experience of using the new RF-CL model as an alternative to the traditional Diamond–Forrester highlights the need for further evaluation in larger cohorts.

To objectively validate the RF-CL model, ICA would need to be performed in all study participants. As such verification of obstructive CAD was not feasible, our findings should be interpreted with caution. Accordingly, this study has several limitations.

One of the promising non-invasive methods for assessing the hemodynamic significance of coronary stenoses is computed tomography-derived fractional flow reserve (FFR-CT) [16]. Its use may reduce the need for invasive coronary imaging and has been associated with an increased risk of cardiovascular mortality when FFR-CT is <0.80 (HR 3.0, 95% CI 1.33–6.76; *p* = 0.008) [17]. However, none of the patients in our cohort underwent FFR-CT assessment, as treating physicians did not consider this investigation to be clinically indicated.

It should also be noted that the evaluation of patients with INOCA is gaining increasing attention. This concept has led to the standardization of diagnostic criteria, including: (1) the presence of symptoms, (2) absence of obstructive coronary artery disease, (3) objective evidence of myocardial ischemia, and (4) evidence of coronary microvascular dysfunction [18]. However, methods for confirming microvascular dysfunction remain limited in routine clinical practice.

Identification of patient groups with a high likelihood of myocardial ischemia may improve selection for stress testing and, in cases of positive results without significant coronary stenosis, facilitate further evaluation of the coronary microcirculation. In our study, ROC analysis demonstrated that the highest probability of a positive stress test was observed in men with an RF-CL value of 19.5%, while in the overall cohort the threshold was 15%. In women, the area under the curve was not acceptable, likely due to the low number of positive stress test results, which represents an additional limitation of the study.

Furthermore, the absence of a clearly defined diagnostic algorithm for suspected INOCA in current Russian clinical guidelines may contribute to the limited use of advanced imaging modalities, such as CT myocardial perfusion. Although these techniques are increasingly adopted, they were rarely used in our cohort and were performed in only one patient. Analysis of our institutional database revealed that only 20 such studies were performed over the past year in patients with suspected INOCA.

In the future, broader application of the RF-CL model may be complemented by the integration of laboratory biomarkers (e.g., ApoB and high-sensitivity C-reactive protein) and advanced imaging techniques, including stress cardiac magnetic resonance imaging and positron emission tomography. However, wider implementation of this diagnostic approach will require the development of unified clinical guidelines. We share the view that the RF-CL model may play a substantially broader role in contemporary cardiology practice. At present, the RF-CL model is primarily focused on estimating the probability of obstructive CAD. However, modern management of CCS extends beyond the assessment of anatomical stenosis and increasingly emphasizes overall cardiovascular risk reduction, including lipid-lowering strategies and combination pharmacotherapy. Even after achieving optimal low-density lipoprotein cholesterol levels, residual cardiovascular risk persists, driven by inflammation, metabolic factors, and non-obstructive CAD [19]. Therefore, an ideal diagnostic stratification approach should be closely linked to clinical decision-making, including combination treatment strategies [20]. In this context, the RF-CL model may serve not only as a diagnostic tool but also as part of a broader risk management strategy in patients with CCS, particularly in those classified as having low or intermediate clinical likelihood yet remaining at residual risk.

## Figures and Tables

**Table 1:** Patients’ baseline characteristics.

Variable	Men (n = 100)	Women (n = 122)	*p*-Value
Age, years	63.92 ± 11.26	65.56 ± 9.3	*p* = 0.25
Smoking, n (%)	38 (38)	13 (10.7)	*p* < 0.001
Body mass index, kg/m^2^	28.73 ± 4.04	29.3 ± 4.91	*p* = 0.34
Dyslipidemia, n (%)	93 (93)	109 (89.3)	*p* = 0.48
Hypercholesterolemia, n (%)	54 (54)	51 (41.8)	*p* = 0.07
Lipid-lowering therapy, n (%)	54 (54)	51 (41.8)	*p* = 0.07
Hypertension, n (%)	88 (88)	113 (92.6)	*p* = 0.26
T2DM, n (%)	17 (17)	20 (16.4)	*p* = 0.9
Atrial fibrillation, n (%)	21 (21)	8 (6.5)	*p* = 0.001
C2-C3b CKD, n (%)	12 (12)	22 (18)	*p* = 0.55
NYHA class I-II, n (%)	6 (6)	5 (4.1)	*p* = 0.51
Peripheral artery disease, n (%)	42 (42)	43 (35.2)	*p* = 0.3
RF-CL, % (IQR)	17 (11–27)	6 (4–10)	*p* < 0.001
Moderate, n (%)	64 (64)	12 (9.8)	*p* < 0.001
Low, n (%)	30 (30)	74 (60.7)	*p* < 0.001
Very low, n (%)	6 (6)	36 (29.5)	*p* < 0.001

T2DM—type 2 diabetes mellitus, CKD—chronic kidney disease, RF-CL—Risk Factor-Weighted Clinical Likelihood. Values were presented as the mean ± standard deviation, as n (%) or as % (IQR).

**Table 2:** Clinical presentation in men and women with suspected CCS.

Clinical Presentation of CCS	Men (n = 100)	Women (n = 122)	*p*-Value
Typical angina, n (%)	21 (21)	21 (17.2)	*p* = 0.47
Atypical angina, n (%)	24 (24)	38 (31.1)	*p* = 0.24
Non-anginal chest pain, n (%)	31 (31)	32 (26.2)	*p* = 0.43
Dyspnea, n (%)	44 (44)	73 (59.8)	*p* = 0.02

Values were presented as n (%).

**Table 3:** Stress test variants in men and women with different CCS presentations.

Stress Test Modalities	Men (n = 100)	Women (n = 122)	*p*-Value
SPECT, n (%)	13 (13)	14 (11.5)	*p* = 0.12
Stress echocardiography, n (%)	55 (55)	82 (66.7)	*p* = 0.06
Exercise ECG testing, n (%)	45 (45)	37 (30.3)	*p* = 0.025

SPECT—single-photon emission computed tomography. Values were presented as n (%).

**Table 4:** Characteristics of patients with positive and negative stress test results in men and women.

Variable	Men (n = 78)			Women (n = 93)		
	Positive (n = 21)	Negative (n = 57)	*p*-Value	Positive (n = 7)	Negative (n = 86)	*p*-Value
Age, years	67 ± 9.06	62.4 ± 11.8	*p* = 0.75	61.1 ± 9.04	65.63 ± 8.69	*p* = 0.72
Smoking, n (%)	4 (19)	26 (47.4)	*p* = 0.03	0 (0)	11 (12.8)	*p* = 0.3
Body mass index, kg/m^2^	29.02 ± 2.93	28.89 ± 4.3	*p* = 0.98	29.7 ± 3.7	29.43 ± 5.34	*p* = 0.97
Dyslipidemia, n (%)	18 (85.7)	55 (96.5)	*p* = 0.11	6 (85.7)	77 (89.5)	*p* = 0.56
Hypertension, n (%)	19 (90.4)	49 (86)	*p* = 0.7	5 (71.4)	79 (91.9)	*p* = 0.08
T2DM, n (%)	5 (23.8)	9 (15.8)	*p* = 0.5	1 (14.3)	17 (19.8)	*p* = 1
Peripheral artery disease, n (%)	8 (38.1)	24 (42.1)	*p* = 0.8	2 (28.6)	29 (33.7)	*p* = 1
Atrial fibrillation, n (%)	5 (23.8)	9 (15.8)	*p* = 0.5	0 (0)	5 (5.8)	*p* = 1
RF-CL, % (IQR)	27 (19–44)	17 (11–20)	*p* < 0.001	6 (4–10)	6 (3.5–11.5)	*p* = 0.85
Moderate, n (%)	18 (85.7)	32 (56.1)	*p* = 0.02	2 (28.6)	6 (7)	*p* = 0.1
Low, n (%)	3 (14.3)	21 (36.8)	*p* = 0.1	2 (28.6)	56 (65.1)	*p* = 0.09
Very low, n (%)	0 (0)	4 (7)	*p* = 0.18	3 (42.9)	24 (27.9)	*p* = 0.4

T2DM—type 2 diabetes mellitus, RF-CL—Risk Factor-Weighted Clinical Likelihood. Values were presented as the mean ± standard deviation, as n (%) or as % (IQR).

**Table 5:** Diagnostic performance of the RF-CL model based on ROC analysis.

	Cut-Off (%)	Sensitivity	Specificity	AUC	*p*-Value
Men	19.5	0.667	0.662	0.723	*p* = 0.002
Women	6.5	0.429	0.571	0.479	*p* = 0.852
All patients	15	0.714	0.72	0.737	*p* < 0.001

AUC—area under the curve.

## Data Availability

The raw data supporting the conclusions of this article will be made available by the corresponding author on reasonable request.

## Abbreviations

The following abbreviations are used in this manuscript:

CAD	Coronary artery disease
CCS	Chronic coronary syndrome
DF	Diamond–Forrester
RF-CL	Risk Factor-Weighted Clinical Likelihood
ICA	Invasive coronary angiography
CKD	Chronic kidney disease
T2DM	Type 2 diabetes mellitus
CHF	Chronic heart failure
SPECT	Single-photon emission computed tomography
CT	Computed tomography
FFR	Fractional Flow Reserve
INOCA	Ischemia with Non-Obstructive Coronary Arteries
FFR-CT	Computed tomography-derived fractional flow reserve

## References

[1] Knuuti J., Wijns W., Saraste A., Capodanno D., Barbato E., Funck-Brentano C., Prescott E., Storey R.F., Deaton C., Cuisset T. (2020). 2019 ESC Guidelines for the Diagnosis and Management of Chronic Coronary Syndromes. Eur. Heart J..

[2] Vrints C., Andreotti F., Koskinas K.C., Rossello X., Adamo M., Ainslie J., Banning A.P., Budaj A., Buechel R.R., Chiariello G.A. (2024). 2024 ESC Guidelines for the Management of Chronic Coronary Syndromes. Eur. Heart J..

[3] Solola Nussbaum S., Henry S., Yong C.M., Daugherty S.L., Mehran R., Poppas A. (2022). Sex-Specific Considerations in the Presentation, Diagnosis, and Management of Ischemic Heart Disease: JACC Focus Seminar 2/7. J. Am. Coll. Cardiol..

[4] Barbarash O.L., Karpov Y.A., Panov A.V., Akchurin R.S., Alekyan B.G., Alekhin M.N., Aronov D.M., Harutyunyan G.K., Belenkov Y.N., Boytsov S.A. (2024). 2024 Clinical practice guidelines for Stable coronary artery disease. Russ. J. Cardiol..

[5] Roth G.A., Mensah G.A., Johnson C.O., Addolorato G., Ammirati E., Baddour L.M., Barengo N.C., Beaton A.Z., Benjamin E.J., Benziger C.P. (2020). Global Burden of Cardiovascular Diseases and Risk Factors, 1990–2019: Update From the GBD 2019 Study. J. Am. Coll. Cardiol..

[6] Douglas P.S., Hoffmann U., Patel M.R., Mark D.B., Al-Khalidi H.R., Cavanaugh B., Cole J., Dolor R.J., Fordyce C.B., Huang M. (2015). Outcomes of Anatomical versus Functional Testing for Coronary Artery Disease. N. Engl. J. Med..

[7] Douglas P.S., Nanna M.G., Kelsey M.D., Yow E., Mark D.B., Patel M.R., Rogers C., Udelson J.E., Fordyce C.B., Curzen N. (2023). Comparison of an Initial Risk-Based Testing Strategy vs Usual Testing in Stable Symptomatic Patients With Suspected Coronary Artery Disease: The PRECISE Randomized Clinical Trial. JAMA Cardiol..

[8] Hemal K., Pagidipati N.J., Coles A., Dolor R.J., Mark D.B., Pellikka P.A., Hoffmann U., Litwin S.E., Daubert M.A., Shah S.H. (2016). Sex Differences in Demographics, Risk Factors, Presentation, and Noninvasive Testing in Stable Outpatients With Suspected Coronary Artery Disease: Insights From the PROMISE Trial. JACC Cardiovasc. Imaging.

[9] Winther S., Schmidt S., Mayrhofer T., Bøtker H., Hoffmann U., Douglas P., Wijns W., Bax J., Nissen L., Lynggaard V. (2020). Incorporating Coronary Calcification Into Pre-Test Assessment of the Likelihood of Coronary Artery Disease. J. Am. Coll. Cardiol..

[10] Gorog D. (2026). Great Debate: The New Risk Factor-Weighted Clinical Likelihood Model Is Useful to Estimate the Initial Pre-Test Probability of Obstructive Coronary Artery Disease in Individuals with Suspected Chronic Coronary Syndromes. Eur. Heart J..

[11] Jespersen L., Hvelplund A., Abildstrøm S., Pedersen F., Galatius S., Madsen J., Jørgensen E., Kelbæk H., Prescott E. (2012). Stable Angina Pectoris with No Obstructive Coronary Artery Disease Is Associated with Increased Risks of Major Adverse Cardiovascular Events. Eur. Heart J..

[12] Van Der Bijl P., Gulati M., Saraste A., Marwick T., Kwong R., Blankstein R., Nieman K., Sengupta P., Van Rosendael A., Knuuti J. (2025). Contemporary, Non-Invasive Imaging Diagnosis of Chronic Coronary Artery Disease. Lancet.

[13] Shaw L.J., Bairey Merz C.N., Pepine C.J., Reis S.E., Bittner V., Kelsey S.F., Olson M., Johnson B.D., Mankad S., Sharaf B.L. (2006). Insights from the NHLBI-Sponsored Women’s Ischemia Syndrome Evaluation (WISE) Study: Part I: Gender Differences in Traditional and Novel Risk Factors, Symptom Evaluation, and Gender-Optimized Diagnostic Strategies. J. Am. Coll. Cardiol..

[14] Rajkumar C.A., Foley M.J., Ahmed-Jushuf F., Nowbar A.N., Simader F.A., Davies J.R., O’Kane P.D., Haworth P., Routledge H., Kotecha T. (2023). A Placebo-Controlled Trial of Percutaneous Coronary Intervention for Stable Angina. N. Engl. J. Med..

[15] Collet C., Mahendiran T., Fearon W.F., Mizukami T., Munhoz D., Pijls N.H.J., Tonino P.A.L., Barbato E., Piroth Z., Sreckovic M. (2026). Fractional Flow Reserve-Guided Percutaneous Coronary Intervention versus Medical Therapy for Stable Coronary Artery Disease: Long-Term Results of the FAME 2 Trial. Nat. Med..

[16] Matteucci A., Massaro G., Mamas M.A., Biondi-Zoccai G. (2021). Expanding the Role of Fractional Flow Reserve Derived from Computed Tomography (FFRCT) for the Non-Invasive Imaging of Patients with Coronary Stents: Rise of the Machines?. Eur. Radiol..

[17] Fairbairn T.A., Mullen L., Nicol E., Lip G.Y.H., Schmitt M., Shaw M., Tidbury L., Kemp I., Crooks J., Burnside G. (2025). Implementation of a National AI Technology Program on Cardiovascular Outcomes and the Health System. Nat. Med..

[18] Ong P., Camici P.G., Beltrame J.F., Crea F., Shimokawa H., Sechtem U., Kaski J.C., Bairey Merz C.N. (2018). Coronary Vasomotion Disorders International Study Group (COVADIS). International Standardization of Diagnostic Criteria for Microvascular Angina. Int. J. Cardiol..

[19] Di Fusco S.A., Volpe M., Nardi F., Matteucci A., Aquilani S., Marino G., Aiello A., Colivicchi F. (2025). Reducing LDL-Cholesterol to Very Low Levels: Sailing Between Established Benefits and Potential Risks. High Blood Press. Cardiovasc. Prev..

[20] Di Fusco S.A., Aquilani S., Spinelli A., Alonzo A., Matteucci A., Castello L., Imperoli G., Colivicchi F. (2023). The Polypill Strategy in Cardiovascular Disease Prevention: It’s Time for Its Implementation. Prog. Cardiovasc. Dis..

